# The Ferment Treatment of Cancer and Tuberculosis

**Published:** 1899-09

**Authors:** 


**The Ferment Treatment of Cancer and Tuberculosis.** By HORACE MANDERS, M.D. Pp. xix., 251. London: The Rebman Publishing Co., Limited. 1898.

Giving a clear exposition of a fresh development of therapeutics, this book brings forward certain new and important facts. It explains thoroughly the treatment of various diseases by "pure ferments," and the *rationale* of it; but it is disfigured by too much padding *de omnibus rebus et multis aliis.* If we had been spared thirty pages on cells and bacteria, twenty-eight on the pathology of tuberculosis, with the symptoms and modes of infection, and a vast amount of information easily obtainable from text-books, but having no connection with the subject, the interest of the book would have been greater, for in its main thesis it is sound.

The chief practical result so far of bacteriology has been serum-treatment. Dr. Manders shows that there is another, the ultimate importance of which may be far greater. Living yeasts act as phagocytes and destroy pathological bacilli. He shows that absolutely pure yeasts can be introduced into the human body, and can multiply there for a time without injury, causing leucocytosis, converting some of the sugar derivatives into alcohol (which he has measured so far as excreted by the lungs) and destroying certain pathological bacteria. Thus in phthisis the cells may be found in the expectoration enclosing degenerate bacilli. Of these important facts he gives convincing proofs; and, in addition, he believes that the cells, having done their work, are of nutrient value, supplying nuclein, succinic acid, and certain mineral substances.

Dr. Manders employs the pure yeast or backérine as a hypodermic injection, as a powder upon ulcers, and occasionally by the mouth, rectum, or as a spray for diphtheritic throats. The hypodermic dose is 1 c.c. at intervals of a week or longer.

There is a sharp reaction followed by improved appetite, and in phthisis a diminution of bacilli and night-sweats with general improvement. Yeast seems to inhibit altogether the growth of the typhoid bacillus *in vitro*, and it has been used with some benefit in typhoid fever. In future papers the writer promises abstracts of the cases of phthisis, typhoid, and septicæmia in which he has used it with advantage. We hope his materials will be put together in a better form. The method deserves careful examination and testing.

